# Synergy of ammonium chloride and moisture on perovskite crystallization for efficient printable mesoscopic solar cells

**DOI:** 10.1038/ncomms14555

**Published:** 2017-02-27

**Authors:** Yaoguang Rong, Xiaomeng Hou, Yue Hu, Anyi Mei, Linfeng Liu, Ping Wang, Hongwei Han

**Affiliations:** 1 Michael Grätzel Center for Mesoscopic Solar Cells, Wuhan National Laboratory for Optoelectronics, School of Optical and Electronic Information, Huazhong University of Science and Technology, Wuhan 430074, China

## Abstract

Organometal lead halide perovskites have been widely used as the light harvester for high-performance solar cells. However, typical perovskites of methylammonium lead halides (CH_3_NH_3_PbX_3_, X=Cl, Br, I) are usually sensitive to moisture in ambient air, and thus require an inert atmosphere to process. Here we demonstrate a moisture-induced transformation of perovskite crystals in a triple-layer scaffold of TiO_2_/ZrO_2_/Carbon to fabricate printable mesoscopic solar cells. An additive of ammonium chloride (NH_4_Cl) is employed to assist the crystallization of perovskite, wherein the formation and transition of intermediate CH_3_NH_3_X·NH_4_PbX_3_(H_2_O)_2_ (X=I or Cl) enables high-quality perovskite CH_3_NH_3_PbI_3_ crystals with preferential growth orientation. Correspondingly, the intrinsic perovskite devices based on CH_3_NH_3_PbI_3_ achieve an efficiency of 15.6% and a lifetime of over 130 days in ambient condition with 30% relative humidity. This ambient-processed printable perovskite solar cell provides a promising prospect for mass production, and will promote the development of perovskite-based photovoltaics.

## Abstract (web summary)

The commercialization of solar cells based on hybrid perovskites requires challenges of device stability and scalable production to be addressed. Rong *et al*. report ambient-processed printable mesoscopic perovskite solar cells with a lifetime of over 130 days in ambient air with 30% relative humidity.

Organometal trihalide perovskites of methylammonium lead halides (CH_3_NH_3_PbX_3_, X=Cl, Br, I) have attracted intensive research attention in the field of energy conversion due to their distinct optical and electronic properties, such as high absorption coefficient, high charge carrier mobility, long diffusion length and low defect density^1^,^2^,^3^. The power conversion efficiency (PCE) of perovskite solar cells (PSCs) has increased from 3.81% in 2009 (ref. 4) to a certified 22.1% in 2016, making it the fastest-advancing photovoltaic technology to date^5^,^6^,^7^,^8^. Besides the high efficiency, PSCs demonstrate dominant cost advantages compared with conventional silicon-based solar cells, because of low-cost raw materials and simple solution-manufacturing process. However, the degradation issue of typical perovskite CH_3_NH_3_PbI_3_ associated with moisture challenges the mass production and further applications of this new-born technology^9^,^10^,^11^.

Recently, abundant work both experimental and theoretical has been devoted to investigating the degradation of CH_3_NH_3_PbI_3_ associated with moisture and develop ambient stable devices. Kamat *et al*. reported that hydrated perovskite phases of CH_3_NH_3_PbI_3_·H_2_O and (CH_3_NH_3_)_4_PbI_6_·2H_2_O will form during the initial degradation process of CH_3_NH_3_PbI_3_ due to humidity^12^. While the monohydrate phase can be fully reversed by exposing the hydrated perovskite to a dry environment, the irreversible decomposition of the dihydrate phase will lead to the complete degradation of CH_3_NH_3_PbI_3_ (ref. 13). Various approaches have been developed to improve the stability of PSCs against moisture, such as perovskite composition optimization^14^,^15^, interface modification^16^,^17^ and device encapsulation. Compared with typical three-dimensional perovskites, two-dimensional layered perovskites have exhibited greatly improved stability against humidity^18^. However, the device performance still cannot compete with that of conventional perovskite-based devices^19^. To attain stability together with high performance, Huang and co-workers^20^ retained CH_3_NH_3_PbI_3_ as the light absorber but bonded crosslinkable silane molecules with hydrophobic functional groups on fullerene to make the fullerene-based electron transport layer and the whole device highly water-resistant. Snaith and co-workers^21^ and Menna and co-workers^22^ replaced the most widely used hole transport layer of 2,2′,7,7′-tetrakis(*N*,*N*-di-p-methoxy-phenylamine)-9,9′-spirobifluorene (spiro-OMeTAD) with organic functionalized single-walled carbon nanotubes, and remarkably enhanced the device water resistance. Bella *et al*.^23^ developed a multifunctional fluorinated photopolymer-coating technique, boosting the device efficiency to nearly 19% and device lifetime to over 6 months.

In achieving high-performance PSCs, the perovskite film quality such as morphology, crystallinity and defect density of the perovskite grains play a significant role. Various deposition methods such as antisolvent treatment^24^, sequential two-step method^25^ and vapour-assisted process^26^ and so on have been developed to attain uniform and homogeneous perovskite thin films for mesoscopic or planar heterojunction PSCs. However, to depress the moisture-assisted degradation of perovskite CH_3_NH_3_PbI_3_ during thin-film-formation process, these techniques usually require inert atmosphere or high vacuum conditions to restrain the environmental humidity, which usually couples with intensive energy-consumption issues and accordingly is incompatible with industrial production. The additive-assisted one-step solution-processing method is considered to meet the requirements for facile film-formation method as well as improved device stability^16^,^27^,^28^. Particularly, optimizing these methods under ambient conditions and correlating them with device performance will lead to a critical understanding of perovskite crystallization kinetics in ambient air, and provide a simple and effective way to fabricate efficient PSCs with improved moisture tolerance. Successful transfer from artwork in the glove box to application modules in ambient air will be an important milestone in the development of such low-cost photovoltaic technology.

Here we employ an additive of ammonium chloride (NH_4_Cl) to tune the crystallization of perovskite CH_3_NH_3_PbI_3_ in ambient air. It has been reported that NH_4_Cl is able to retard the crystallization of CH_3_NH_3_PbI_3_ and act as a binder to interconnect separated CH_3_NH_3_PbI_3_ crystals^27^,^29^. However, the kinetics behind this process is still unknown. In our work, benefited from the microthick triple-layer scaffold^30^,^31^, the phase transformation of perovskite crystals in the presence of ammonium and moisture is slowed down and can be monitored. We find a synergy of NH_4_Cl and moisture on the crystallization of perovskite CH_3_NH_3_PbI_3_. Through forming an intermediate of CH_3_NH_3_X·NH_4_PbX_3_(H_2_O)_2_ (X=I or Cl), the crystallization of CH_3_NH_3_PbI_3_ is first retarded. The moisture in ambient air accelerates the removal of ammonium, and thus induces the transition of intermediate to perovskite phase. On the basis of a triple-layer PSC architecture, the devices fabricated under ambient conditions show a PCE of 15.6%, and ambient air lifetime of over 130 days with performance maintaining ∼96.7% of the initial value.

## Results

### Compositional and crystallographic characterizations

**Figure 1:**
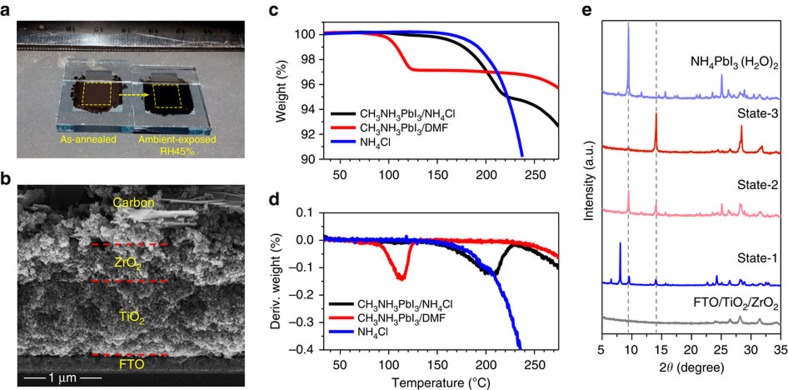
Compositional and crystallographic characterization. (**a**) Digital images (from the glass side) of the as-annealed (crystallization step-1: 100 °C for 10 min) and ambient-exposed (crystallization step-2: relative humidity 45% for 36 h) precursor-infiltrated triple-layer scaffold. The active area of the device is marked with a yellow rectangle. (**b**) Cross-sectional SEM image of perovskite-infiltrated TiO_2_/ZrO_2_/Carbon triple layer. The triple layer was deposited by screen-printing techniques, and the perovskite absorber was deposited using an NH_4_Cl-containing precursor solution and a moisture-induced crystallization process. (**c**,**d**) TGA results for the samples of CH_3_NH_3_PbI_3_/NH_4_Cl (red), CH_3_NH_3_PbI_3_/DMF (grey) and pure NH_4_Cl (blue). (**e**) X-ray diffraction patterns of the scaffold (FTO/TiO_2_/ZrO_2_), precursor-infiltrated scaffold in state-1 (as-infiltrated), state-2 (solvent-evaporated) and state-3 (annealed), and NH_4_PbI_3_(H_2_O)_2_-infiltrated scaffold.

To achieve high-performance printable PSCs, it is critical to control the crystallization of perovskite absorbers in the triple-layer scaffold of mesoporous TiO_2_ (m-TiO_2_) electron-collecting layer, ZrO_2_-insulating layer and carbon counter electrode layer. The precursor was prepared by dissolving 1.0 M PbI_2_, 1.0 M CH_3_NH_3_I and 1.0 M NH_4_Cl in N,N-Dimethylformamide (DMF), and simply dripped on the carbon layer of the scaffold. After a short-term thermal annealing of 10 min at 100 °C, the precursor was dried and homogeneously distributed in the active area of the scaffold, as shown in Fig. 1a. The active area is defined by the overlap section of the TiO_2_, ZrO_2_ and carbon layer as highlighted. Interestingly, the as-annealed sample was light brown, which is different from the colour of typical CH_3_NH_3_PbI_3_ absorber^5^,^9^,^32^, indicating that an intermediate phase rather than a pure perovskite phase may exist at this state (*vide infra*). After being exposed to ambient air with relative humidity (RH) of 45%, the colour of the sample turned dark brown. This distinct exterior variation implied a significant conversion from an intermediate to perovskite phase that occurred during the ambient exposure, leading to homogeneous distribution of CH_3_NH_3_PbI_3_ absorbers in the scaffold, as shown in Fig. 1b.

According to the thermal gravimetric analysis (TGA) results of CH_3_NH_3_PbI_3_/DMF, CH_3_NH_3_PbI_3_/NH_4_Cl and pure NH_4_Cl (Fig. 1c,d), the thermal annealing process (defined as crystallization step-1) resulted in the evaporation of solvent in the precursor along with partial decomposition of NH_4_Cl, since sample of CH_3_NH_3_PbI_3_/DMF shows distinct weight loss between 100 and 120 °C, while the sample of CH_3_NH_3_PbI_3_/NH_4_Cl only shows tiny weight loss below 150 °C. Pure NH_4_Cl or NH_4_Cl/CH_3_NH_3_PbI_3_ began to lose weight until the temperature increased to over 200 °C. During the thermal annealing process at 100 °C, NH_4_Cl decomposed and released NH_3_ gas, which has been confirmed by annealing the sample in a Petri dish with pH test paper (Supplementary Fig. 1). The residual NH_4_Cl after thermal annealing process was detected using the Glow Discharge Optical Emission Spectrometry analysis (Supplementary Fig. 2). Thus, after crystallization step-1, an intermediate with the composition of CH_3_NH_3_I·PbI_2_·*x*NH_4_Cl (*x*<1) may exist in the scaffold.

The structural information of the intermediate and phase transition was collected using X-ray diffraction measurements as shown in Fig. 1e. To avoid the interference caused by carbon layer, the precursor was infiltrated in the scaffold of m-TiO_2_/ZrO_2_. The as-infiltrated sample (state-1) showed distinct solvent-associated peaks between 2*θ*=5° and 10° (ref. 33). After exposure to ambient air at RT for several minutes, the solvent of DMF in the precursor evaporated (state-2) and the peaks at 9.46° and 14.10° significantly enhanced, which can be assigned to (101) of NH_4_PbI_3_(H_2_O)_2_ and (110) of CH_3_NH_3_PbI_3_ crystal^34^,^35^. It was proposed that NH_4_^+^ can form perovskite NH_4_PbI_3_ with a calculated bandgap of 1.53 eV (ref. 36). However, since the calculated Goldschmidt tolerance factor of this compound lies close to the stability limit, it may exist in quasi-one-dimensional structures (Supplementary Fig. 3) especially in the presence of moisture^34^,^37^. For the thermal annealed sample (state-3), the peak at 9.46° largely reduced while the peak at 14.10° significantly enhanced, indicating that a significant transformation from NH_4_PbI_3_(H_2_O)_2_ to CH_3_NH_3_PbI_3_ occurred during the thermal annealing process, in which a large amount of NH_3_ was released. Through identifying the composition and structure of the intermediate, it can be concluded that, in the presence of NH_4_^+^ and moisture, the [PbI_6_]^4-^ octahedral will first form [PbI_3_]^−^ double chains and then bond together by NH_4_^+^ and water molecules, which retards the formation of CH_3_NH_3_PbI_3_. The intermediate is considered a mixture of CH_3_NH_3_X·NH_4_PbX_3_(H_2_O)_2_ (X=I or Cl). As the amount of ammonium reduced during thermal annealing, CH_3_NH_3_X will immediately combine with PbX_2_ and form perovskite CH_3_NH_3_PbX_3_.

### Device performance under ambient conditions

To gently convert the intermediate to perovskite CH_3_NH_3_PbI_3_ in the triple-layer scaffold, ambient exposure process (defined as crystallization step-2) was carried out under ambient conditions with RH of 35–65%. According to the Grotthuss mechanism^38^, the water molecule is able to remove one proton from ammonium. The deprotonation of ammonium in CH_3_NH_3_X·NH_4_PbX_3_(H_2_O)_2_ leads to the formation of NH_3_, and HI or HCl. Since ammonia is extremely volatile and soluble in water, this moisture-induced reaction will continue until ammonium in the intermediate completely decomposes. Significantly, a reversible intercalation of NH_3_ in the perovskite crystal lattice was also observed in such triple-layer scaffold-hosted CH_3_NH_3_PbI_3_ (Supplementary Movie 1). Thus, once NH_4_^+^ in the intermediate turns to NH_3_, it will release, not trap in the scaffold, facilitating the crystallization of CH_3_NH_3_PbI_3_.

**Figure 2:**
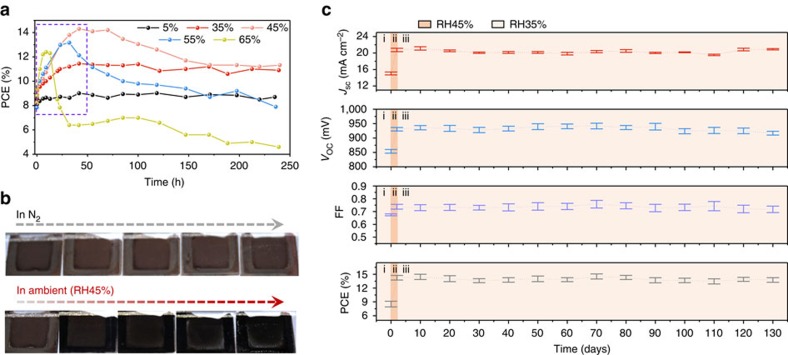
Comparison of the device performance under ambient conditions. (**a**) PCE variations of as-annealed devices stored under ambient conditions with RH5–65%. The significant improvement in efficiency is marked with a purple rectangle. (**b**) Digital images (from the glass side) of the as-annealed devices stored in inert (N_2_) and ambient atmosphere (RH45%). (**c**) Variations of *J*_SC_, *V*_OC_, FF and PCE of devices upon fabricating and ageing under ambient condition without encapsulation ((i) device fabrication except the ambient exposure, RH35%; (ii) ambient exposure, RH45%; (iii) long-term storage, RH35%). Error bars represent s.d. calculated from four devices prepared at the same conditions.

Figure 2a presents the evolution of device performance during this process. Surprisingly, the intermediate CH_3_NH_3_X·NH_4_PbX_3_(H_2_O)_2_ containing devices still showed initial efficiencies of 8–9%, indicating that NH_4_^+^ did not completely restrain but only partially inhibit the crystallization of perovskite. Generally, when the as-annealed devices were exposed to ambient air, the efficiencies increased dramatically in the first 10–48 h, and then remained stable (RH35%), decreased slightly (RH45% and RH55%), or decayed sharply (RH65%). This remarkable enhancement in device performance indicated the formation of high-quality perovskite absorber in the triple-layer scaffold under ambient conditions, since the evolution occurred with the colour change of the device (from glass side) from light brown to dark brown (Fig. 2b). On the contrary, for the device stored in N_2_, no distinguishable colour change can be observed, indicating that such transition needs to be induced by ambient air. It has been confirmed that the ambient condition can accelerate the decomposition of NH_4_Cl (Supplementary Fig. 4). Considering that the higher RH values caused faster efficiency enhancement, it can be concluded that the moisture in ambient air plays a critical role for the crystallization of CH_3_NH_3_PbI_3_, and controlling the RH for device fabrication might lead to high-performance devices. At the same time, the degradation occurred over RH45% after reaching peak efficiencies should be effectively prevented.

Considering that the ambient condition of RH45% that led to the highest efficiency and that devices at RH35% presented almost no degradation, devices were fabricated at RH35% (i), exposed to RH45% (ii) and stored at RH35% (iii), as shown in Fig. 2c. After reaching an efficiency of ∼14%, the devices were stored in the dark at room temperature (RT, 25 °C) without encapsulations. In a period of 130 days, all the four photovoltaic parameters of short-circuit current density (*J*_SC_), open-circuit voltage (*V*_OC_), fill factor (FF) and PCE stayed constant. Particularly, the PCE maintained 96.7% of the initial value after over 130-day storage. These results demonstrate that the penetration of moisture into the perovskites is effectively prevented or inhibited. In the case of RH<35%, moisture in the ambient air will not combine with the perovskite absorbers in the scaffold and cause degradation. This agrees with the result obtained by the as-annealed device stored in RH35%. At a low RH, the moisture neither penetrates into the intermediate in the scaffold nor accomplishes the crystallization of perovskite CH_3_NH_3_PbI_3_. Thus, the efficiency of the device only slightly increased to ∼11% and stayed unchanged during the ambient exposure.

### *In situ* characterizations of intermediate during ambient exposure

**Figure 3:**
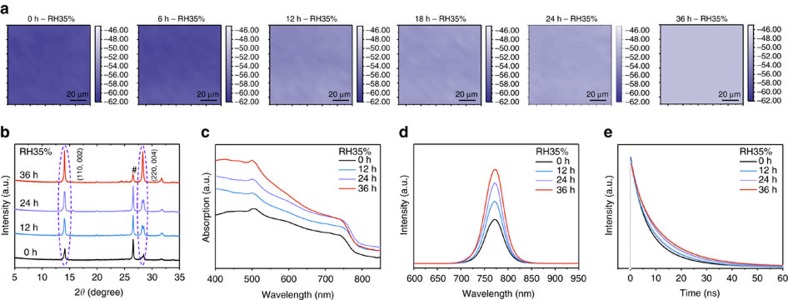
*In situ* characterization of intermediate evolution in the scaffolds during ambient exposure. (**a**) *In situ* FTIR mapping measurement results of intermediate infiltrated in the ZrO_2_ layer during ambient exposure (RH35%). The scanning area is 100 μm × 100 μm, and the intensity is calculated by the peak area between 2,890 and 3,340 cm^−1^, which corresponds to the vibration of N–H. (**b**) X-ray diffraction patterns of intermediate infiltrated in TiO_2_/ZrO_2_/Carbon triple layer during ambient exposure (RH35% for 36 h). # represents the peak of carbon. The (110)/(002) and (220)/(004) peaks of CH_3_NH_3_PbI_3_ are also marked. (**c**) Ultraviolet–visible (UV–vis) spectra of intermediate infiltrated in m-TiO_2_ layer during ambient exposure (RH35% for 36 h). (**d**) Steady-state PL spectra of intermediate infiltrated in the ZrO_2_ layer during ambient exposure (RH35% for 36 h). (**e**) Time-resolved PL spectra of intermediate infiltrated in the ZrO_2_ layer during ambient exposure (RH35% for 36 h).

In order to investigate the transformation during the ambient exposure, *in situ* characterizations of intermediate-infiltrated m-TiO_2_ layer, ZrO_2_ layer or TiO_2_/ZrO_2_/Carbon triple layer were performed. Although the moisture-induced crystallization process in the triple-layer scaffold, which is significantly influenced by the layer thickness and microstructure, could be different from that in a single layer, the results still provide an insight into the transformation of the intermediate and correlate it with device performance. The Fourier transform infrared spectroscopy (FTIR) measurements were performed with intermediate hosted by FTO/ZrO_2_ scaffold. As exposed to the ambient air, the peak area between 2,890 and 3,340 cm^−1^ that corresponds to the vibration of N–H significantly decreased (Supplementary Fig. 5), indicating the reduction of NH_4_^+^ or CH_3_NH_3_^+^ in the sample. An area of 100 μm × 100 μm on the sample was selected to perform an *in situ* FTIR mapping measurement, as shown in Fig. 3a. The intensity was calculated by the peak area (2,890–3,340 cm^−1^). In the first 6 h, no distinct variation was observed, but after 12 h the signal intensity began to decrease. Further exposed to ambient air for 24 and 36 h, the signal for N–H vibration kept decreasing. For each single image during this process, the tiny variation indicated homogeneous distribution of CH_3_NH_3_X·NH_4_PbX_3_(H_2_O)_2_ and CH_3_NH_3_PbI_3_, and the moisture-induced transition occurred continuously once started. It was also found that the transition induced by moisture originated from the edges of the devices and then continuously extended to the whole active area of the device (Supplementary Fig. 6). This implied that the moisture in ambient air penetrated into the mesoporous scaffold from the boundaries, and the combination of water molecule and ammonium occurred as a chain reaction. Of course, water molecule can also remove the proton from ammonium of CH_3_NH_3_PbI_3_, leading to the decomposition of the perovskite^39^. Thus, excessive exposure to high humid (RH55-65%) ambient air has led to degradation of device performance.

X-ray diffraction measurements were performed with intermediate-infiltrated triple layer to characterize the perovskite growth during the ambient exposure, and the results are presented in Fig. 3b. For the 0 h exposed sample, peaks attributed to perovskite structure CH_3_NH_3_PbI_3_ are quite weak, although the (002), (110), (004) and (220) peaks of CH_3_NH_3_PbI_3_ in tetragonal (*I*4/*mcm*) symmetry can be distinguished. After ambient exposure, the intensities of these peaks and their combinations of (002)/(110) and (004)/(220) both significantly increased, indicating enlarged grain size and increased crystallinity. Remarkably, the intensity of the (004)/(220) peak reached a comparable intensity to (002)/(110) peak after 36 h exposure. This indicates a crystal growth with preferential orientation along the [110] direction during the moisture-induced crystallization process. At the same time, it should be noted that the peaks of perovskite hydrates or PbI_2_ have not been found^12^,^13^, indicating that no water intrusion or perovskite decomposition occurred during the ambient exposure. We propose that there is a balance between the water in the intermediate/perovskite-infiltrated scaffold and in ambient exposure. It is much easier for NH_4_PbI_3_ to form hydrates compared with CH_3_NH_3_PbI_3_ in ambient air. Thus, at a low RH such as 35%, the water molecules only combine with NH_4_^+^ in the intermediate, but will not negatively influence the perovskite crystals. Along with the phase transformation, the morphology of the perovskite absorber in the triple-layer scaffold would also slightly evolve. Since the perovskite crystal growth was significantly templated by scaffold without any capping layer, the morphology of the perovskite absorber will not change drastically during the ambient exposure process.

During the ambient exposure, the absorption of intermediate-infiltrated m-TiO_2_ layer also significantly enhanced (Fig. 3c). Considering all the samples show an onset at 750 nm, which matches with the result of typical tetragonal perovskite phase of CH_3_NH_3_PbI_3_, we suppose NH_4_^+^ only partially retard the crystallization of the perovskite absorber, since NH_4_PbX_3_(H_2_O)_2_ shows a colour of light yellow and no absorption in the range of 450–900 nm (Supplementary Fig. 7). *In situ* photoluminescence (PL) spectra of ZrO_2_/intermediate were measured to correlate the quality of the perovskite absorber in the scaffold with the moisture-induced crystallization. Upon exposure, the peak intensity at 773 nm significantly increased, but no distinguishable peak shift can be observed in the steady-state PL spectra (Fig. 3d). For the time-resolved PL spectra, the charge carrier lifetime slightly increased from 11.50 to 13.33 ns (Fig. 3e).

## Discussion

**Figure 4:**
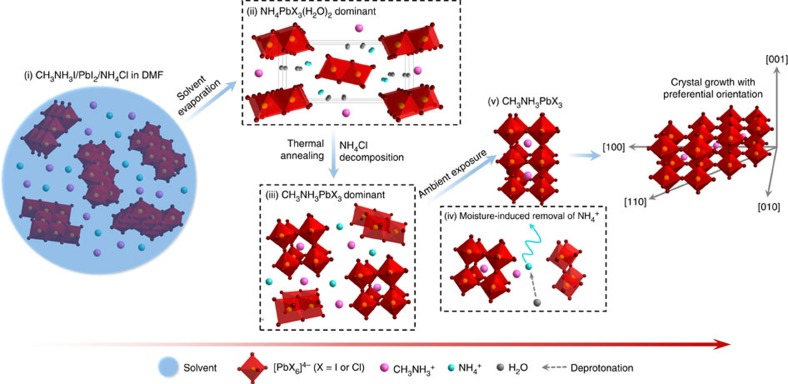
Schematic view of the crystal growth process of perovskite CH_3_NH_3_PbX_3_ in the presence of ammonium and moisture. (i) CH_3_NH_3_I, PbI_2_ and NH_4_Cl dissolved in DMF. (ii) As the solvent evaporated, NH_4_^+^ and PbX_3_^−^ formed dominantly an ammonium lead trihalide dehydrate of NH_4_PbX_3_(H_2_O)_x_. (iii) During thermal annealing, NH_4_PbX_3_(H_2_O)_x_ released NH_3_ and combined with CH_3_NH_3_^+^, partially transforming to perovskite CH_3_NH_3_PbX_3_. (iv) Moisture induced the removal of NH_4_^+^ and facilitated the (v) crystallization of CH_3_NH_3_PbX_3_. At the final stage, CH_3_NH_3_PbX_3_ crystals grew along the [110] direction.

Similar results that moisture can assist the growth of perovskite films and improve the film quality, grain size and carrier lifetime have been reported^40^,^41^. More directly, water was added to the perovskite precursor to tune the crystallization of CH_3_NH_3_PbI_3−*x*_Cl_*x*_ (ref. 42). However, in our case, the crystallization of perovskite CH_3_NH_3_PbI_3_ was determined by the synergy of NH_4_Cl and moisture. We proposed a potential mechanism in view of microscopic dynamics as shown in Fig. 4. In the first stage, the solvent molecules intercalated in PbI_2_ when CH_3_NH_3_I, PbI_2_ and NH_4_Cl dissolved in DMF^33^,^43^. As the solvent evaporated, NH_4_^+^ and PbX_3_^−^ formed an ammonium lead triiodide dehydrate of NH_4_PbX_3_(H_2_O)_2_. After a short-term thermal annealing, NH_4_PbX_3_(H_2_O)_2_ released NH_3_ and combined with CH_3_NH_3_^+^, partially transforming to perovskite CH_3_NH_3_PbI_3_. Such fast decomposition led to uncontrolled crystallization of CH_3_NH_3_PbI_3,_ producing a poor crystallinity of the resulting perovskite polycrystalline layer with a tiny amount of residual NH_4_PbX_3_(H_2_O)_2_ and CH_3_NH_3_I distributed in the mixed intermediate phase. Because of the presence of NH_4_^+^ in/between the crystals, the crystallization of CH_3_NH_3_PbI_3_ was retarded. During the ambient exposure process, moisture induced the removal of NH_4_^+^ and facilitated the crystallization of CH_3_NH_3_PbI_3_. At the final stage, the crystal growth with preferential orientation along the [110] direction was considered to be caused by chlorine in the system^44^. X-ray photoelectron spectroscopy measurements that were performed after thermal annealing and after ambient exposure confirmed that a trace amount of chlorine existed in the resulting perovskite-infiltrated triple-layer scaffold (Supplementary Fig. 8).

To fabricate high-performance devices and correlate the intermediate transformation with device performance, the RH was carefully controlled at 45% for ambient exposure and then decreased to <35% for storage. The current density–voltage (*J–V*) curves of the as-annealed device during 0–36 h ambient exposure is presented in Supplementary Fig. 9. It can be found that the enhancement in PCE was mainly attributed to the improved *J*_SC_, which dramatically increased from 15.16 to 20.67 mA cm^−2^. A modest increase in *V*_OC_ and FF negligibly contributed to the improved device performance. The enhanced photocurrent density was confirmed by incident photon-to-electron conversion efficiency (IPCE) spectra (Supplementary Fig. 10), for which the integrated current values showed an error of less than 5% compared with those measured in *J–V* measurements. Since the 1 μm-thick m-TiO_2_ in the triple-layer device can provide sufficient contact area for the perovskite absorber, we proposed that the electron injection efficiency was a constant. The transition from CH_3_NH_3_X·NH_4_PbX_3_(H_2_O)_2_ to CH_3_NH_3_PbI_3_ mainly contributed to the enhancement in light-harvesting efficiency and charge collection efficiency for IPCE. After 36 h exposure, the IPCE increased dramatically from 60 to over 80% in the range of 400–650 nm with peak close to 90%. These increases in IPCE and *J*_SC_ were in accordance with the enhanced absorption spectra as presented in Fig. 3c.

**Figure 5:**
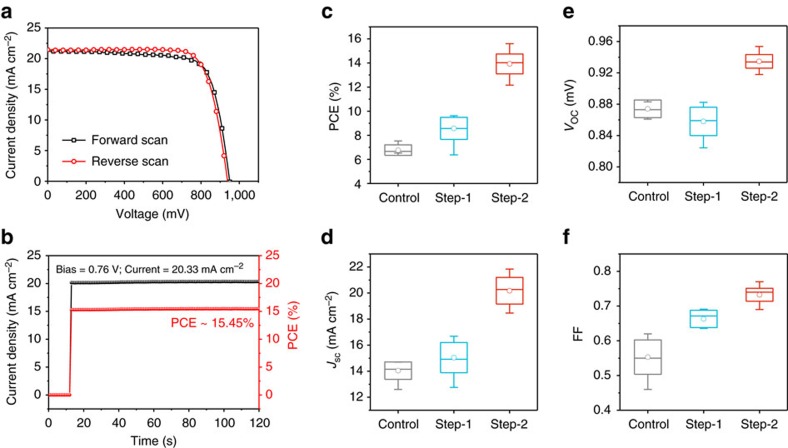
Performance of the champion device and distributions of device photovoltaic parameters. (**a**) *J–V* curves of the champion device using NH_4_Cl-containing precursor and moisture-induced crystallization process measured by reverse scan (RS) and forward scan (FS) with a scan rate of 100 mV s^−1^. RS: *V*_OC_=0.94 V, *J*_SC_=21.45 mA cm^−2^, FF=0.77, PCE=15.60%; FS: *V*_OC_=0.95 V, *J*_SC_=21.20 mA cm^−2^, FF=0.75, PCE=15.17%. (**b**) Stabilized output of the device measured under a bias of 0.76 V, presenting a current density of 20.33 mA cm^−2^ and a PCE of 15.45%. (**c**–**f**) Histograms of PCE, *J*_SC_, *V*_OC_ and FF of devices fabricated using conventional drop-casting method with precursor of PbI_2_ and CH_3_NH_3_I in DMF (control), and moisture-induced crystallization method (step-1 for thermal annealing, step-2 for ambient exposure) with precursor of PbI_2_, CH_3_NH_3_I and NH_4_Cl in DMF. The data are represented as a standard box plot where the box range, median line and circle dot are defined by the s.d., middle value and average value. The whiskers represent the outlier values with a coefficient of 1.5. The results are summarized from 30 devices.

A champion efficiency of 15.60% with *J*_SC_ of 21.45 mA cm^−2^, *V*_OC_ of 0.94 V and FF of 0.77 was achieved for such ambient-processed printable PSCs, as shown in Fig. 5a. The *J–V* curves were measured at a scan rate of 100 mV s^−1^. Comparing the *J–V* curves measured with reverse and forward scans, a slight hysteresis phenomenon existed, mainly showing tiny variation in FF. Stabilized output measurement was performed to evaluate the accurate PCE of the device. Under a bias of 0.76 V, the current sharply increased to 20.33 mA cm^−2^ once exposed to 1 sun illumination, demonstrating an efficiency of 15.45% (Fig. 5b). This efficiency is quite close to the values obtained in the *J–V* measurements.

On the basis of the previous discussions, 30 devices were fabricated using the optimal two-step crystallization-involved method. The photovoltaic parameters of the device for step-1 and step-2 are compared, and the results obtained with traditional one-step method are plotted as a reference (Fig. 5c–f and Table 1). The control devices show average PCE of 6.77%, and the poor device performance is mainly due to the relatively low average *J*_SC_ of 14.04 mA cm^−2^ and FF of 0.55. Incomplete pore-filling of perovskite absorber limited the light-harvesting and charge transportation. For the devices fabricated in step-1, similar efficiencies were obtained though *V*_OC_ and FF varied. The low *J*_SC_ was caused by the retarded crystallization of perovskite absorbers, not the incomplete pore-filling. After ambient exposure as step-2, the device efficiency significantly improved to an average value of 13.92% along with increased average *J*_SC_ of 20.21 mA cm^−2^, *V*_OC_ of 0.935 V and FF of 0.73. Detailed distributions of the photovoltaic parameters are presented in Supplementary Fig. 11.

In summary, we report a synergy effect of NH_4_Cl and moisture on perovskite crystallization in printable PSCs. The NH_4_Cl and moisture-assisted deposition method provides a facile way to accomplish complete pore-filling of perovskite absorbers in the mesoporous triple-layer scaffold, which plays a significant role in attaining efficient and stable printable PSCs. A comprehensive understanding of the mechanism that NH_4_^+^ retarded and a moisture-induced crystallization process is built. Correspondingly, the devices based on CH_3_NH_3_PbI_3_ achieve an average PCE of 13.92% with a champion efficiency of 15.60% and a lifetime of over 130 days in ambient air with RH of 35%. This design and strategy suggest promising prospects for further mass production of perovskite-based photovoltaics and will significantly promote the development of perovskite-based photovoltaics.

## Methods

### Materials

Unless stated otherwise, all materials were purchased from Sigma-Aldrich or Acros Organics and used as received. CH_3_NH_3_I was synthesized and purified according to literature procedures^45^. The perovskite precursor solution was prepared by dissolving PbI_2_ (461 mg, 1.0 M) and CH_3_NH_3_I (159 mg, 1.0 M) with/without NH_4_Cl (53.5 mg, 1.0 M) in DMF (1.0 ml), and stirred at 70 °C for 30 min. The TiO_2_, ZrO_2_ and carbon pastes were prepared as previously reported^30^.

### Device fabrication

Unless stated otherwise, the whole device fabrication process was carried out under ambient conditions (RH35%). The FTO-coated glass substrates (Tec15, Pilkington) were first etched by laser and cleaned by ultrasonication with detergent, deionized water, acetone and ethanol. A compact TiO_2_ layer was then deposited on the patterned substrates by aerosol spray pyrolysis at 450 °C using a titanium diisopropoxide bis(acetylacetonate) solution diluted in ethanol (1:39, volume ratio). After cooling to RT (25 °C), a 1 μm-thick mesoporous TiO_2_ layer, a 1 μm-thick ZrO_2_ spacer layer and a 10 μm-thick carbon layer were screen-printed on the substrates layer by layer. The TiO_2_ and ZrO_2_ layers were sintered at 450 °C for 30 min, and the carbon layer was sintered at 400 °C for 30 min, forming the mesoporous triple-layer-based scaffold. After cooling to RT, the precursor solution with or without NH_4_Cl was infiltrated into the triple layer by drop-casting via the edge of the carbon layer. After annealing at 100 °C for 10 min (crystallization step-1: thermal annealing), the as-annealed samples were exposed to ambient conditions with RH of 5–65% (crystallization step-2: ambient exposure). The ambient exposure was performed in an environmental chamber (Vötsch, C4-180), which can provide an ambient condition with RH of 10–98% (deviation: ±1 to ±3%), or in a dry-air (RH≤5%) filled glove box. For the fabrication of the best-performing devices exhibiting a PCE of over 15%, crystallization step-2 was performed under RH45% for 48 h, and all the other procedures were carried out under RH35%.

### Characterization

The cross-sectional scanning electron microscopy (SEM) image of the perovskite-infiltrated triple layer was obtained by a field-emission scanning electron microscope (Nova NanoSEM 450, FEI). The TGA measurements were carried out at a heating rate of 10 °C min^−1^ under nitrogen or ambient airflow (Pyris1 TGA, PerkingElmer). The *in situ* characterizations of FTIR, X-ray diffraction, UV–vis and PL spectra were performed at RH35% in ambient air. To simulate the conditions in device fabrication, the samples were prepared by depositing intermediate in TiO_2_ layer, ZrO_2_ layer or TiO_2_/ZrO_2_/carbon triple layer on FTO glass substrates. The FTIR measurements (Vertex 70, Bruker) were performed with the FTO/ZrO_2_/intermediate. The X-ray diffraction measurements were performed on an X-ray diffractometer (X'pert PRO, Cu Kα radiation, 40 kV) and detected through the intermediate side of FTO/TiO_2_/ZrO_2_/carbon/intermediate. The UV–vis spectra were obtained with FTO/TiO_2_/intermediate on a spectrophotometer (LabRAM HR800, Horiba). The steady-state and time-resolved PL measurements of FTO/ZrO_2_/intermediate were carried out on a fluorescence spectrometer (DeltaFlex, Horiba). Glow Discharge Optical Emission Spectrometry measurement was performed on GD-Profiler 2 (Horiba). Photocurrent density–voltage (*J–V*) curves were characterized with a Keithley 2400 sourcemeter and a Newport solar simulator (model 91192). The power of the simulated light was calibrated to 100 mW cm^−2^ using a Newport Oriel PV reference cell (model 91150 V). The active area of the device is ∼0.8 cm^2^, and a black mask with a circular aperture (0.126 cm^2^) was applied for *J–V* measurements. The *J–V* testing was performed with both reverse and forward scan directions at 100 mV s^−1^ (sweep delay time of 100 ms). No preconditioning protocol was used before the characterization. The IPCE spectra were measured using a 150 W xenon lamp (Oriel) fitted with a monochromator (Cornerstone 74004) as a monochromatic light source. Calibration with the Oriel Si detector was carried out before IPCE measurements. Unless stated otherwise, all the measurements were carried out at RH35% and RT, which was controlled by the air conditioner at the testing centre.

### Data availability

The data supporting the findings of this study are available from the corresponding author upon request.

## Additional information

**How to cite this article:** Rong, Y. *et al*. Synergy of ammonium chloride and moisture on perovskite crystallization for efficient printable mesoscopic solar cells. *Nat. Commun.* **8,** 14555 doi: 10.1038/ncomms14555 (2017).

**Publisher's note:** Springer Nature remains neutral with regard to jurisdictional claims in published maps and institutional affiliations.

## Supplementary Material

Supplementary Information Supplementary Figures and Supplementary References

Supplementary Movie 1 The reversible intercalation of NH3 in the perovskite crystal lattice. The perovskite absorber of CH3NH3PbI3 was infiltrated in the TiO2/ZrO2/Carbon triple-layer scaffold to fabricate a printable mesoscopic perovskite solar cell. The cell was exposed to NH3·H2O vapor. After about 4 seconds, the color of the cell began to turn from dark brown to white, indicating the intercalation of NH3 in the crystal lattice of CH3NH3PbI3. When NH3·H2O vapor was removed, the cell immediately recovered to its initial state, indicating the intercalation of NH3 in the perovskite crystal lattice was reversible.

## Figures and Tables

**Table 1:** Photovoltaic parameters for printable PSCs fabricated with conventional drop-casting method (control) and moisture-induced crystallization method.

**Sample method**	***V***_**OC**_** (V)**	***J***_**SC**_ **(mA cm**^**−2**^**)**	**FF**	**PCE (%)**
Control	0.874±0.011	14.04±0.67	0.553±0.049	6.77±0.44
Step-1	0.858±0.018	15.04±1.16	0.663±0.025	8.57±0.92
Step-2	0.935±0.009	20.21±1.03	0.733±0.019	13.92±0.82

FF, fill factor; PCE, power conversion efficiency; PSC, perovskite solar cell.

s.d.'s are calculated from 30 cells for each method.

Step-1 for thermal annealing, step-2 for ambient exposure.
