# Supplementary material for: Synergy of ammonium chloride and moisture on perovskite crystallization for efficient printable mesoscopic solar cells
Source: Nat Commun. 2017 Feb 27;8:14555. doi: 10.1038/ncomms14555 (PMC5333356; doi:10.1038/ncomms14555)
Supplement: Supplementary Information — Supplementary Figures and Supplementary References [file ncomms14555-s1.pdf]

(i) (ii) (iii) (iv)

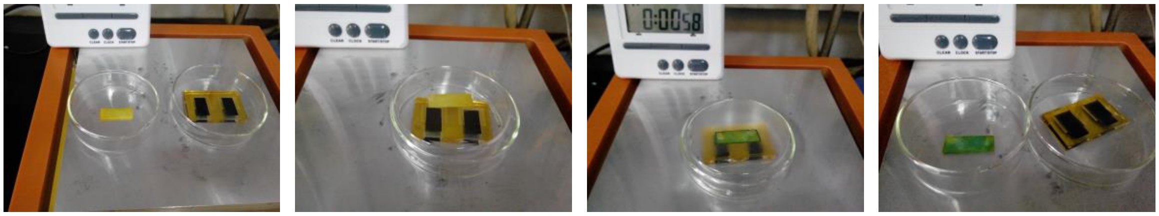

Supplementary Figure 1 | Detection of NH_3 during the thermal annealing process. On a FTO glass substrate, a triple-layer of TiO_2/ZrO_2/Carbon was screen-printed and sintered[1]. Then, 2.5 μl NH_4Cl containing precursor solution was dropped on the edge of the carbon layer. (i) the precursor solution infiltrated TiO_2/ZrO_2/Carbon triple-layer and a PH test paper were placed in a pair of petri dishes; (ii) the petri dishes were annealed at 100 ^{o}C; (iii) The PH test paper in the petri dishes turned blue after 58 s; (iv) the significant color change of the PH test paper indicated the release of NH_3 and decomposition of NH_4Cl in the precursor infiltrated triple-layer during the thermal annealing process.

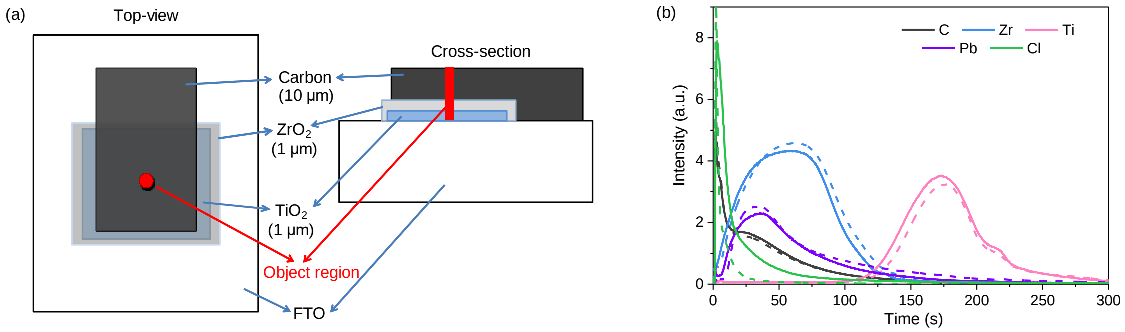

Supplementary Figure 2 | Glow Discharge Optical Emission Spectrometry (GD-OES) analysis. The distributions of different elements in the triple-layer scaffold were characterized by GD-OES measurement. The triple-layer of TiO_2/ZrO_2/Carbon was deposited on a FTO glass substrate. Then NH_4Cl containing precursor was infiltrated in the triple-layer and annealed at 100 ^oC for 10 min. (**a**) the scheme for preparing the samples and corresponding thickness of different layers. (**b**) distributions of carbon (C), zirconium (Zr), titanium (Ti), plumbum (Pb) and chlorine (Cl) along the vertical direction of the intermediate/perovskite infiltrated triple-layer. The solid line represents the results of the as-annealed sample, while the dash line represents the results of the ambient-exposed sample.

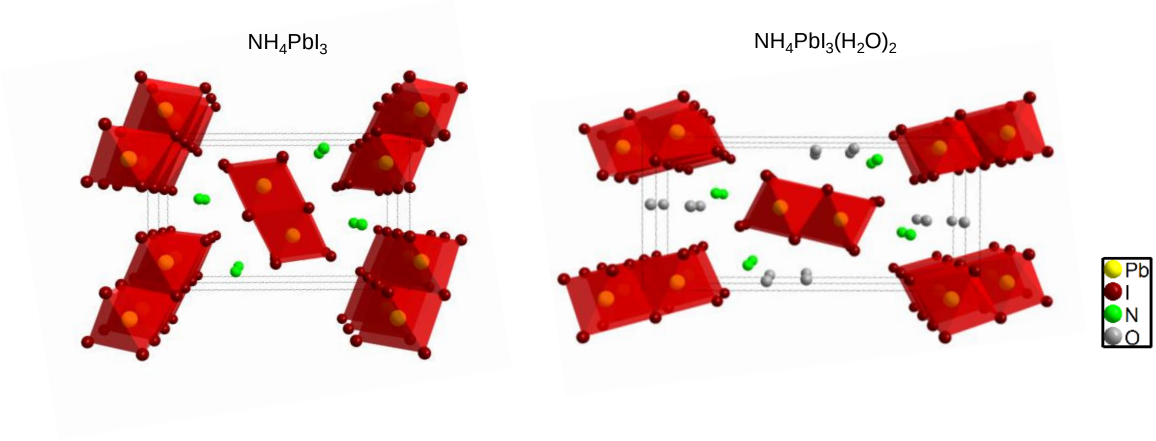

Supplementary Figure 3 | The crystal structure of NH_4PbI_3 and $NH_4PbI_3(H_2O)_2$. NH_4PbI_3 was obtained by solid-state reactions. Each Pb^{2-} ion is coordinated by six I^- ions in a distorted octahedra environment. PbI_6 octahedra are connected by common edges, forming a one-dimensional anion chain[2]. $NH_4PbI_3(H_2O)_2$ contains $[PbI_3]^-$ double chains of edge-sharing PbI_6 octahedra. These chains extend along b and are bonded together by ammonium and water molecules[3].

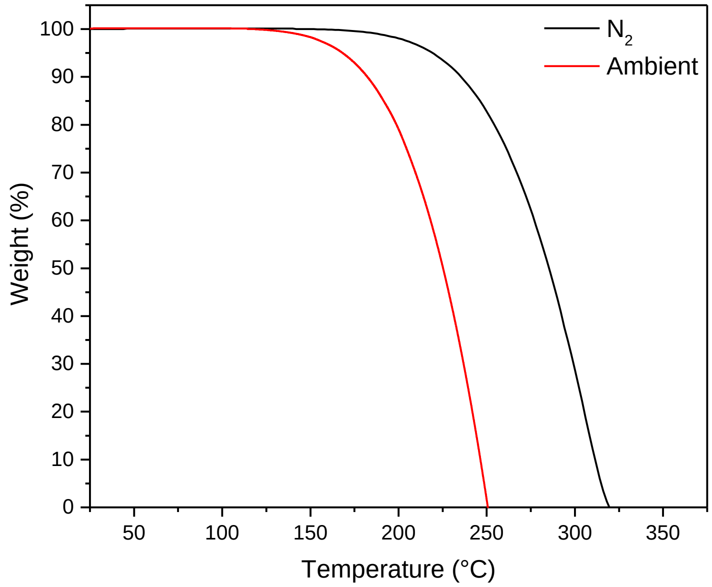

Supplementary Figure 4 | Thermal gravimetric analysis (TGA) of NH_4Cl. TGA measurement of pure NH_4Cl was carried out in nitrogen and ambient air (RH35%). In N_2, NH_4Cl began to decompose at *ca.* 218 °C (5% weight loss). In ambient air, NH_4Cl began to decompose at *ca.* 168 °C.

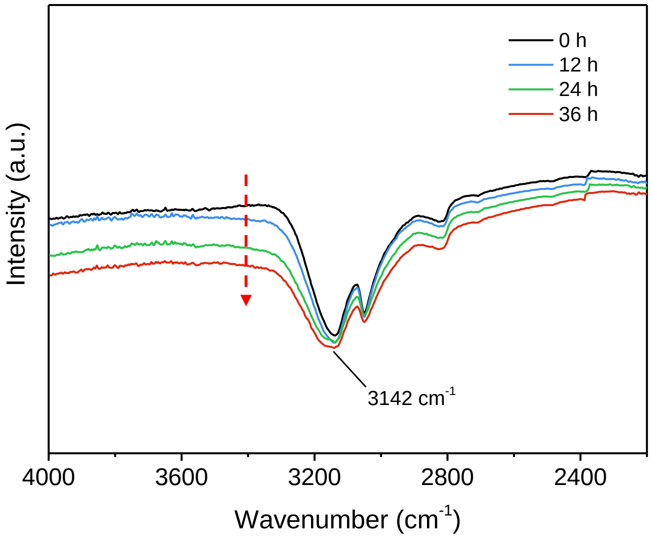

Supplementary Figure 5 | *In-situ* Fourier transform infrared (FTIR) measurements. To investigate the transformation from intermediate $CH_3NH_3X{\cdot}NH_4PbX_3(H_2O)_2$ to perovskite $CH_3NH_3PbI_3$ during ambient exposure process, FTIR spectra of the intermediate hosted by ZrO_2 layer (*ca.* 1.0 μm) were collected at 0, 12, 24 and 36 h under ambient condition (RH35%).

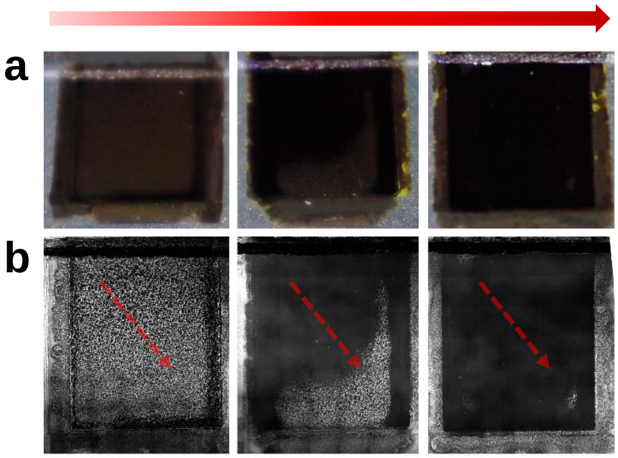

Supplementary Figure 6 | Digital images of the as-annealed devices under ambient condition (RH35%). (**a**) images of the devices (glass side) captured using a digital camera with normal mode. (**b**) images of the devices (glass side) captured using a Confocal Laser Scanning Microscope (CLSM) system with reflection mode.

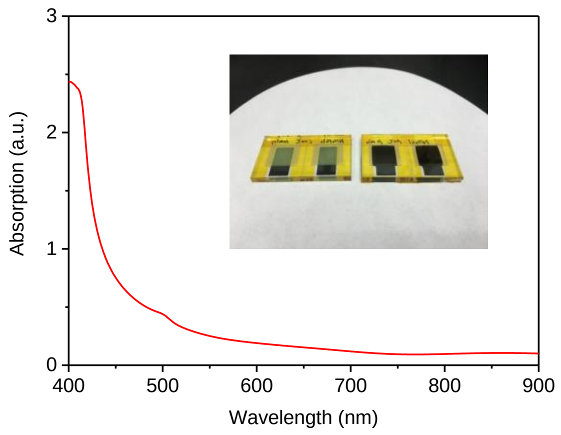

Supplementary Figure 7 | UV-Vis absorption spectrum of $NH_4PbX_3(H_2O)_2$. Sample preparation: a solution of 1.0 M PbI_2 and 1.0 M NH_4I in DMF was deposited in a 1 μm-thick TiO_2 scaffold and annealed at 100 ^oC for 10 min. Inset: the digital images of $NH_4PbI_3(H_2O)_2$ (left) and $CH_3NH_3PbI_3$ (right) infiltrated in the triple-layer.

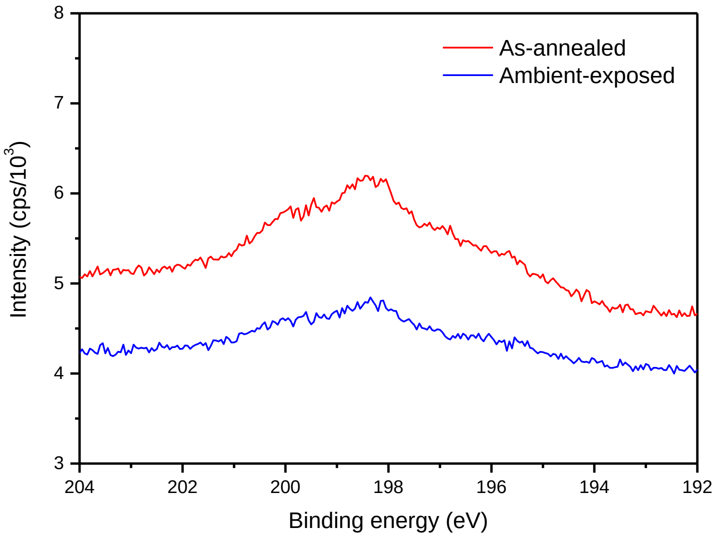

Supplementary Figure 8 | X-ray photoelectron spectroscopy (XPS) results of the as-annealed and ambient-exposed devices. For both devices, only a trace amount of chlorine (Cl 2p) can be detected. This indicates that a large proportion of chlorine has been removed in the form of HCl during thermal annealing. The residual chlorine may exist in the perovskite lattice and influence the growth kinetics of $CH_3NH_3PbX_3$ crystals.

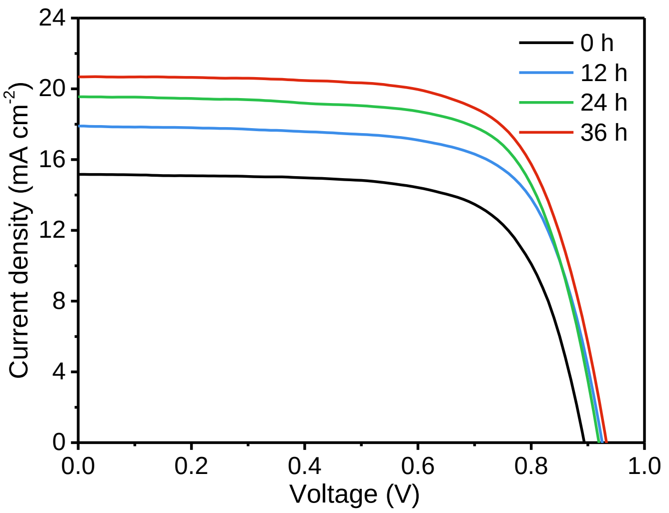

Supplementary Figure 9 | Device performance of the as-annealed device during ambient exposure. Current density - voltage (*J*-*V*) curves of the as-annealed device measured at 0, 12, 24 and 36 h under ambient condition (RH45%).

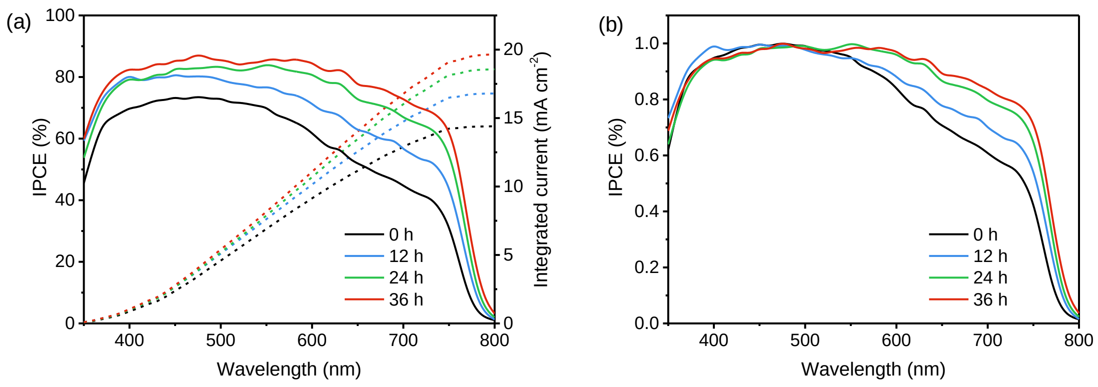

Supplementary Figure 10 | Incident photon to current conversion efficiency (IPCE) of the as-annealed device during ambient exposure. (**a**) IPCE spectra of the as-annealed device measured at 0, 12, 24 and 36 h under ambient condition (RH45%). (**b**) normalized IPCE spectra of the as-annealed device measured at 0, 12, 24 and 36 h under ambient condition (RH45%).

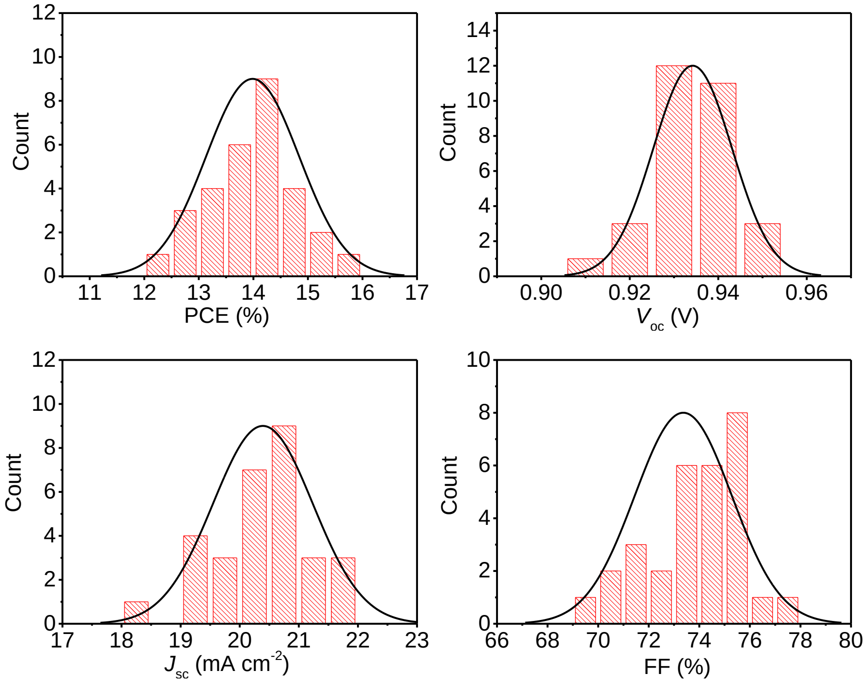

Supplementary Figure 11 | Statistical distributions of the photovoltaic parameters for the device fabricated with optimal procedure. Histograms of *PCE*, V_{OC}, J_{SC} and *FF* of the devices. The thickness of the triple-layer is as below: TiO_2 ~1 μm, ZrO_2 ~1 μm, Carbon ~10 μm. The perovskite crystallization process step-1: annealed at 100 °C for 10 min; step-2: exposed to ambient air (RH45%) for ~ 48 hours.

Supplementary References

1. Ku, Z., Rong, Y., Xu, M., Liu, T. & Han, H. Full Printable Processed Mesoscopic $CH_3NH_3PbI_3/TiO_2$ Heterojunction Solar Cells with Carbon Counter Electrode. *Sci. Rep.* **3**, 3132 (2013).

2. Fan, L.-Q. & Wu, J.-H. NH_4PbI_3. *Acta Cryst. E* **63**, i189-i189 (2007).

3. Bedlivy, D. & Mereiter, K. The structures of potassium lead triiodide dihydrate and ammonium lead triiodide dihydrate. *Acta Cryst. B* **36**, 782-785 (1980).
